# Eye-Tracking Assessment in Patients with Disorders of Consciousness: A Systematic Review

**DOI:** 10.3390/brainsci16060590

**Published:** 2026-05-30

**Authors:** Anna Estraneo, Lorenza Marcello, Francesca Mancino, Alessia De Feo, Andrea Soricelli, Monica Franzese, Carlo Cavaliere

**Affiliations:** 1 Fondazione Don Gnocchi, ONLUS, 83054 Sant’Angelo dei Lombardi, Italy; aestraneo@dongnocchi.it; 2 Laboratory of Neuronal Networks Morphology and Systems Biology, Department o f Mental, Physical Health and Preventive Medicine, University of Campania “Luigi Vanvitelli”, 80138 Naples, Italy; lorenza.marcello@gmail.com; 3 IRCCS SYNLAB SDN, Via E. Gianturco 113, 80137 Naples, Italy; francesca.mancino@synlab.it (F.M.); alessia.defeo@synlab.it (A.D.F.); andrea.soricelli@synlab.it (A.S.); monica.franzese@synlab.it (M.F.)

**Keywords:** disorders of consciousness, eye-tracking, visual pursuit, minimally conscious state, unresponsive wakefulness syndrome, CRS-R

## Abstract

**Highlights:**

**What are the main findings?**

- Eye-tracking detects 2.5× more visual responses than standard clinical observation (46.2% vs. 18.1%), demonstrating its superior sensitivity for identifying covert consciousness signs missed by CRS-R.
- Mirror stimuli achieve the highest detection rates (97% of patients with MCS), followed by person (69%) and object (57%) stimuli, providing immediate practical guidance for optimising clinical assessment protocols.

**What are the implications of the main findings?**

- Advanced VR-based systems achieve high diagnostic accuracy: sensitivity 100% and specificity 88.9% (RPTL-V biomarker), with preliminary prognostic value—62.5% of overt trackers demonstrated command-following at one year.
- High heterogeneity and small sample size of selected studies supported a conditional recommendation for eye-tracking as a supplementary tool alongside CRS-R, not a replacement.

**Abstract:**

Background/Objectives: Disorders of consciousness (DOC), including vegetative state/unresponsive wakefulness syndrome (VS/UWS) and minimally conscious state (MCS), present significant diagnostic challenges. Misdiagnosis rates approach 40%, often due to limitations in detecting subtle behavioural responses through clinical observation alone. Eye-tracking technology offers objective, quantitative assessment of visual behaviours that may reveal covert signs of consciousness. This systematic review aimed to evaluate the diagnostic accuracy of eye-tracking technology compared to the Coma Recovery Scale-Revised (CRS-R) for detecting visual responses and consciousness signs in patients with DOC; to examine stimulus effects; and to assess prognostic value. Methods: A systematic literature search was conducted across SciSpace, Google Scholar, PubMed, and institutional libraries following PRISMA 2020 guidelines. Eligibility criteria specified studies involving patients with prolonged DOC assessed using eye-tracking technology. Data extraction, risk of bias assessment, and GRADE certainty evaluation were conducted systematically. Results: Fifteen studies (n = 4–123 patients; published 2012–2025) were included. Eye-tracking detected visual responses in significantly more patients than clinical observation alone (46.2% vs. 18.1% in one study). Mirror stimuli demonstrated the highest detection sensitivity (97% vs. 69% for person and 57% for object). Affectively salient stimuli elicited stronger tracking responses in patients with MCS (37.3% vs. 29.9–30.6% neutral). Advanced VR-based systems achieved high diagnostic accuracy (sensitivity 100%, specificity 88.9%) with prognostic value (overt tracking predicting 62.5% command-following at one year). GRADE certainty was Low for detection rates and diagnostic discrimination, and Very Low for sensitivity, specificity, and prognostic outcomes. Conclusions: Eye-tracking provides objective, sensitive assessment of visual behaviours in patients with DOC and may reduce misdiagnosis rates, supporting a conditional recommendation for its use as a supplementary assessment tool alongside CRS-R. Methodological heterogeneity, small sample sizes, and absence of blinding limit certainty. Adequately powered, multicentre prospective studies are urgently needed.

## 1. Introduction

Disorders of consciousness (DOC) represent a spectrum of conditions characterised by impaired awareness and wakefulness following severe brain injury [1,2]. The two primary diagnostic categories are vegetative state/unresponsive wakefulness syndrome (VS/UWS), characterised by wakefulness without awareness, and minimally conscious state (MCS), in which patients demonstrate inconsistent but reproducible signs of consciousness [1,2]. Accurate differential diagnosis between these states is critical for prognosis, treatment planning, and ethical decision-making regarding life-sustaining interventions [3,4].

Despite the development of standardised behavioural assessment tools such as the Coma Recovery Scale-Revised (CRS-R) [5], misdiagnosis rates in DOC remain alarmingly high, approaching 40% in some studies [6]. This diagnostic uncertainty stems from multiple factors, including the subtle and fluctuating nature of behavioural responses, motor impairments that mask cognitive abilities, within-day variability in arousal and responsiveness, and the inherent limitations of subjective clinical observation [7,8]. The phenomenon of “covert consciousness”—in which patients possess awareness but cannot reliably demonstrate it through voluntary motor responses—further complicates clinical assessment [1,9,10].

Visual pursuit (VP) and visual fixation (VF) are recognised as cardinal behavioural signs of the minimally conscious state in major diagnostic frameworks, including the CRS-R and the Aspen Workgroup criteria [5]. These visual behaviours are considered among the first signs of emerging consciousness and serve as key diagnostic markers differentiating MCS from VS/UWS [11,12,13,14,15,16]. However, the assessment of VP and VF through clinical observation alone is subject to inter-rater variability, requires specialised training, and may fail to detect subtle or inconsistent responses [1,10].

Eye-tracking technology offers a promising solution to these diagnostic challenges by providing objective, quantitative measurement of gaze behaviour, fixation patterns, and pupillary responses [4,15,17]. Unlike subjective clinical observation, eye-tracking systems can detect and quantify visual behaviours with high temporal and spatial resolution, potentially revealing covert signs of consciousness that escape detection during standard bedside assessment [15,18,19]. Recent technological advances, including wearable eye-tracking devices, virtual reality (VR) integration, and hybrid brain–computer interface (BCI) systems combining eye-tracking with electroencephalography (EEG), have expanded the potential applications of this technology in DOC assessment [20,21,22,23].

Despite growing interest in eye-tracking for DOC assessment, several critical questions remain unanswered. First, the comparative diagnostic accuracy of eye-tracking versus standard clinical assessment has not been systematically evaluated across studies. Second, the influence of stimulus characteristics—including stimulus type (mirror, person, object), affective salience (familiar vs. unfamiliar), and presentation modality (real-world vs. virtual)—on detection rates requires synthesis. Third, the prognostic value of eye-tracking findings for predicting functional recovery and emergence from DOC has not been comprehensively reviewed. Finally, the methodological heterogeneity across studies necessitates critical appraisal to guide future research and clinical implementation.

This systematic review aimed to: (1) evaluate the diagnostic accuracy and sensitivity of eye-tracking technology in detecting VP and VF in patients with DOC; (2) compare eye-tracking with standard CRS-R assessment; (3) examine the influence of stimulus type, affective salience, and presentation modality; (4) assess the prognostic value of eye-tracking findings; (5) explore emerging VR and hybrid BCI technologies; and (6) identify methodological limitations to inform future research.

## 2. Materials and Methods

This systematic review was conducted in accordance with the Preferred Reporting Items for Systematic Reviews and Meta-Analyses (PRISMA) 2020 guidelines [24]. The completed PRISMA 2020 checklist is provided in Supplementary Table S1. The review protocol was developed a priori but was not registered in an international registry such as PROSPERO. The predefined protocol was documented internally to ensure transparency and consistency throughout the review process.

### 2.1. PICO Framework

Population (P): Adult patients (≥18 years) with prolonged DOC (VS/UWS or MCS), including MCS-minus and MCS-plus subcategories, with acquired brain injury of any aetiology (traumatic, hypoxic–ischaemic, stroke, subarachnoid haemorrhage). Chronic phase (>4 weeks post-injury) preferred; acute/subacute not excluded.

Intervention (I): Eye-tracking technology for assessment of visual behaviours, including infrared (stationary or wearable), video-based, VR-integrated, and hybrid EEG-BCI systems. Outcomes: visual pursuit, visual fixation, gaze patterns, and pupillary responses across various stimulus types.

Comparison (C): Standard clinical behavioural assessment, primarily CRS-R; other standardised scales (GCS, WHIM, SMART); clinical observation without technological augmentation; comparison between stimulus types or eye-tracking protocols.

Outcomes (O): Primary: detection rate of VP/VF; diagnostic accuracy (sensitivity, specificity) for differentiating MCS from VS/UWS. Secondary: stimulus type effects; prognostic value for functional recovery; technical feasibility and inter-rater reliability.

### 2.2. Eligibility Criteria

According to predetermined inclusion and exclusion criteria, clinical studies that looked at ET assessment on DOC were included. Inclusion criteria: empirical studies (any design) reporting original data; adult patients (≥18 years) diagnosed with VS/UWS or MCS; any form of eye-tracking technology used for diagnostic or prognostic assessment; quantitative measures of visual behaviour as outcomes (pursuit, fixation, gaze metrics, or derived biomarkers); published in peer-reviewed journals or as preprints (2000–2025); and English language. Exclusion criteria: review articles, editorials, commentaries, and conference abstracts without full-text availability; studies reporting only qualitative or anecdotal observations without quantitative eye-tracking data; studies exclusively involving healthy controls, paediatric populations, or non-DOC neurological conditions; studies using eye-tracking solely for rehabilitation or communication without diagnostic intent; and case reports with fewer than three patients.

### 2.3. Information Sources and Search Strategy

The systematic search was conducted across four databases: SciSpace (n = 100), Google Scholar (n = 55), PubMed (n = 67), and institutional libraries (n = 8). Reference lists of included studies were manually screened for additional relevant publications. Direct retrieval of known relevant studies identified through expert consultation was also performed.

Search terms included combinations of: “eye tracking”, “eye-tracking”, “visual pursuit”, “visual fixation”, “gaze”, “disorders of consciousness”, “vegetative state”, “unresponsive wakefulness syndrome”, “minimally conscious state”, “CRS-R”, “brain injury”, “virtual reality”, and “brain-computer interface”. Complete Boolean search strings for each database are provided in Supplementary File S1. For example, PubMed Search String was: (“eye tracking”[MeSH Terms] OR “eye tracking”[tiab] OR “eye movement”[tiab] OR “gaze tracking”[tiab] OR “visual tracking”[tiab] OR “oculomotor”[tiab] OR “eye-tracking”[tiab] OR “gaze-tracking”[tiab] OR “visual pursuit”[tiab]) AND (“disorder of consciousness”[tiab] OR “disorders of consciousness”[tiab] OR “vegetative state”[tiab] OR “unresponsive wakefulness syndrome”[tiab] OR “minimally conscious state”[tiab] OR “VS/UWS”[tiab] OR “MCS”[tiab] OR “prolonged disorder of consciousness”[tiab] OR “chronic disorder of consciousness”[tiab] OR “coma”[MeSH Terms] OR “persistent vegetative state”[MeSH Terms] OR “consciousness disorders”[MeSH Terms]) AND (“diagnosis”[tiab] OR “diagnostic accuracy”[tiab] OR “detection”[tiab] OR “assessment”[tiab] OR “evaluation”[tiab] OR “classification”[tiab] OR “sensitivity”[tiab] OR “specificity”[tiab] OR “prognosis”[tiab] OR “prognostic”[tiab]).

### 2.4. Study Selection and Data Extraction

**Figure 1:**
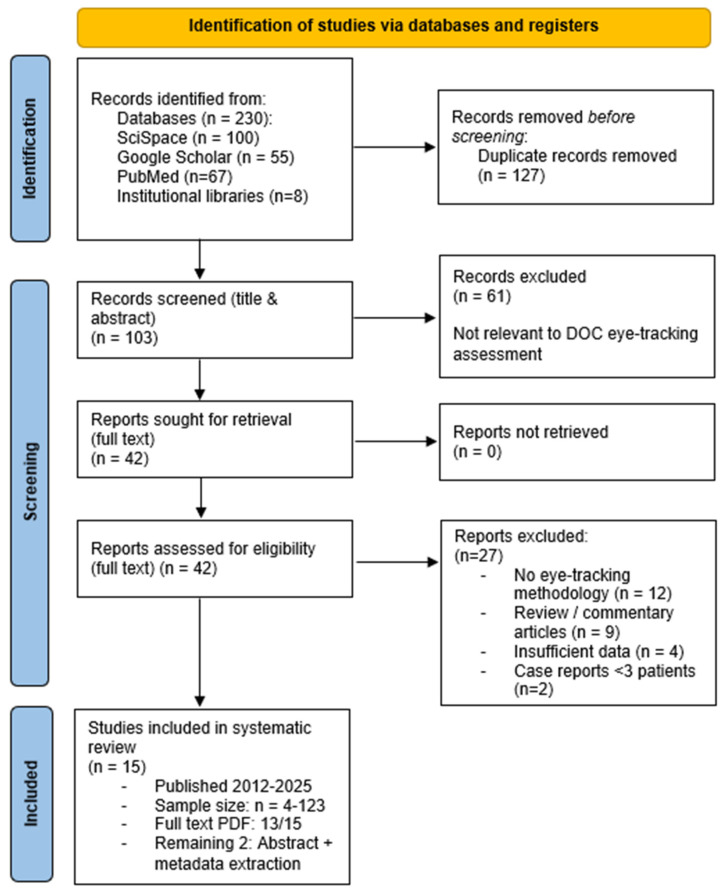
PRISMA Flow Diagram. Study selection process across four database sources (SciSpace, Google Scholar, PubMed, institutional libraries). Records identified: 230; screened: 103; full-text assessed: 42; included: 15. Exclusion reasons at full-text stage: no eye-tracking methodology (n = 12), review/commentary articles (n = 9), insufficient data (n = 4), case reports < 3 patients (n = 2).

After deduplication, two reviewers (A.E. and C.C.) independently screened all titles and abstracts, and subsequently assessed full texts for eligibility. Inter-rater agreement for full-text inclusion was κ = 0.89 (substantial agreement). Disagreements were resolved through consensus discussion; no third reviewer arbitration was required. The PRISMA 2020 flow diagram (Figure 1) details the selection process.

A standardised data extraction form was developed and pilot-tested. Information extracted included: study design, sample size and diagnostic breakdown, eye-tracking device and protocol, stimulus characteristics, detection rates, sensitivity, specificity, AUC, 95% confidence intervals, and key statistical findings. Data extraction was performed from full-text PDFs where available (n = 13) and from abstracts, DOI metadata, and published summaries for remaining studies (n = 2). In particular, full-text PDFs were unavailable for three included studies [23,25]; data for these studies were extracted from published abstracts and DOI metadata only. This constitutes a methodological limitation, as abstract-level extraction is less reliable for complex methodological and statistical variables.

### 2.5. Risk of Bias Assessment

The risk of bias assessment was conducted using a domain-based approach adapted from the Cochrane Risk of Bias tool, applied to observational and non-randomised studies. Each study was evaluated across six bias domains, with each domain rated on a three-level scale: Low for adequate safeguards in place—bias unlikely to affect results; unclear for insufficient information to make a definitive judgement; and High for significant methodological weakness—bias likely present. Inter-rater agreement for bias ratings was κ = 0.82.

### 2.6. Certainty of Evidence

The GRADE (Grading of Recommendations Assessment, Development and Evaluation) framework was applied to five key outcomes [26]. Certainty was assessed across five domains: risk of bias, inconsistency, indirectness, imprecision, and publication bias. Starting certainty for observational studies was Low, with upgrades and downgrades applied per GRADE rules.

## 3. Results

### 3.1. Study Selection and Characteristics

The systematic search identified 103 unique records across all databases. After title and abstract screening, 42 studies were selected for full-text review. Following detailed assessment against eligibility criteria, 15 studies were included in the final systematic review (Figure 1). The included studies were published between 2012 and 2025, with sample sizes ranging from 4 to 123 patients with DOC.

Table 1 presents structured data extraction for all 15 included studies.

### 3.2. Risk of Bias Assessment Findings

Risk of bias was variable across studies. Common limitations included: (1) lack of blinding (assessors not blinded to clinical diagnosis in any study); (2) small sample sizes (median n = 18); (3) heterogeneous reference standards (CRS-R administration varied); (4) incomplete reporting of calibration success rates; and (5) potential selection bias (convenience sampling). Detailed risk of bias assessments are presented in Table 2.

Regarding the sample size, most studies had small to moderate sample sizes (n = 4 to n = 46), with only three studies exceeding 50 participants [29,31,34]. This limits statistical power and generalisability. As for diagnostic criteria, studies using standardised tools (CRS-R) and validated eye-tracking protocols showed lower risk. Blinding of assessors was rarely reported, introducing potential bias in subjective outcome interpretation. Studies with well-defined, standardised eye-tracking protocols showed lower risk. Exploratory and novel technology studies showed moderate risk due to protocol development. Few studies included healthy control groups, limiting ability to establish normative benchmarks. Finally, within-day variability, arousal fluctuations, medication effects, and sensory impairments were inconsistently addressed across studies.

Overall, the majority of studies demonstrated moderate risk of bias, with a few showing low–moderate [14,29] or high risk (several studies with insufficient reporting).

Table 2 summarises the risk of bias assessment across seven domains for all 15 included studies.

**Figure 2:**
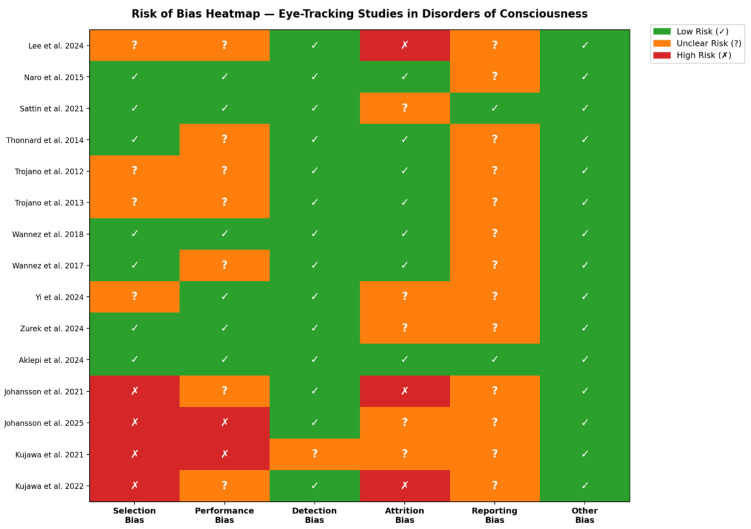
Risk of Bias Heatmap. Colour-coded matrix (Green—Low/Orange—Unclear/Red—High) with ✓ ? ✗ symbols per domain × study [14,15,27,28,29,30,31,32,34,36]. Aklepi 2024 shows the cleanest profile [25]; Kujawa 2021/2022 [33,35] and Johansson 2021/2025 [1,23] have the most high-risk domains.

Figure 2 presents the assessment as a heatmap.

### 3.3. Synthesis of Findings

#### 3.3.1. Detection of Visual Pursuit and Fixation

**Figure 3:**
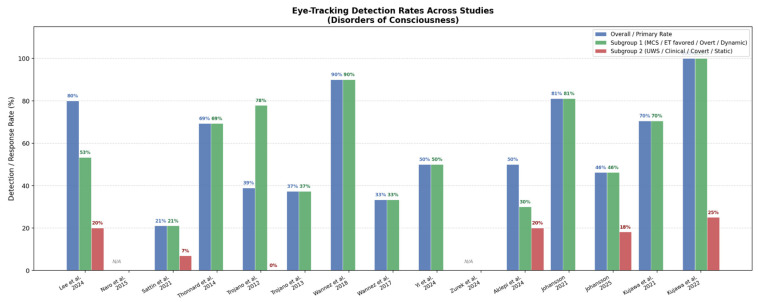
Eye-Tracking Detection Rates Across Studies. Grouped bar chart showing overall + two subgroup rates per study [1,14,15,23,25,27,28,29,30,31,32,33,34,35,36]. Wannez 2018 [32] (90%) and Kujawa 2022 [35] dynamic (100%) show the highest rates, while Sattin 2021 [34] (21%) and Trojano 2013 [28] (37%) the lowest.

Eye-tracking technology demonstrated high sensitivity for detecting visual pursuit and fixation across multiple studies [30,31,34,35]. Trojano et al. (2012) demonstrated that patients with MCS exhibited on-target fixations 67.8% of the time compared to 32.2% in patients with VS/UWS (χ^2^ *p* < 0.001) [27]. The superiority of objective eye-tracking over clinical observation was dramatically demonstrated by Johansson et al. (2025), who found that wearable eye-tracking detected responses in 46.2% of patients (6/13) compared to 18.1% (2/11) detected by CRS-R visual subscore alone, representing a 2.5-fold increase in detection rate (χ^2^(1) = 41.486, *p* < 0.001) [1]. Figure 3 presents detection rates across studies.

#### 3.3.2. Stimulus Type Effects

Mirror stimuli demonstrated consistent superiority across multiple studies. Thonnard et al. (2014) reported that 97% of patients with MCS tracked their own reflection, compared to 69% tracking a person and 57% tracking an object [29]. Affectively salient stimuli (photographs of close relatives) elicited stronger tracking responses in patients with MCS (37.3%) compared to neutral stimuli (29.9–30.6%). For affective salience: χ^2^(1) = 6.8, *p* = 0.009, and d = 0.42 [95% CI: 0.11–0.73]. This effect was not observed in patients with VS/UWS, suggesting that affective processing may be a marker of minimal consciousness.

#### 3.3.3. Objective Eye-Tracking vs. Clinical Assessment

Lee et al. (2024) identified one patient with positive RPTL biomarker responses not detected by CRS-R, demonstrating that eye-tracking can reveal covert consciousness signs missed by behavioural scales [14]. Zurek et al. (2024) compared the MCSD test with the CRS-R in 46 patients and found that eye-tracking provided additional diagnostic information beyond CRS-R alone [15].

#### 3.3.4. Advanced Technologies: VR and Hybrid BCI Systems

Lee et al. (2024) developed a VR-based system using an HTC Vive head-mounted display with integrated infrared pupil tracking, achieving RPTL-V (Repetitive Pupillary Light Reflex in Virtual Reality) sensitivity of 100% and specificity of 88.9% (preprint, not peer-reviewed) [14]. Yi et al. (2024) developed a hybrid BCI system integrating EEG and eye-tracking, achieving ∼88.9% average accuracy for multimodal consciousness assessment [36]. Aklepi et al. (2024) demonstrated that immersive VR stimuli revealed covert tracking responses in patients with TBI not detected with standard stimuli [25].

#### 3.3.5. Prognostic Value

**Figure 4:**
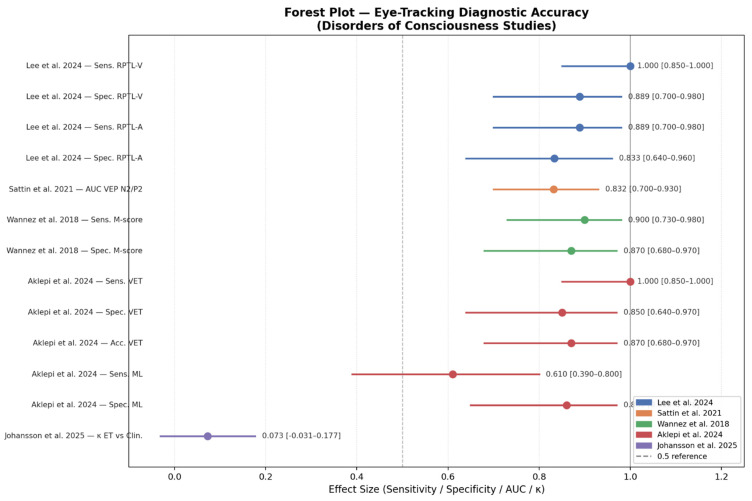
Forest Plot of Eye-Tracking Detection Rates and Diagnostic Accuracy. Plot sensitivity, specificity, AUC, and κ with 95% CIs for 5 studies reporting quantitative diagnostic accuracy [1,14,30,35,36]. Most metrics cluster between 0.83 and 1.00; Johansson 2025 [1] κ = 0.073 is notably low.

Lee et al. (2024) found that overt tracking on the RPTL biomarker predicted command-following at one year in 62.5% of cases (n = 8 overt trackers), providing preliminary evidence for the prognostic utility of eye-tracking [14]. Figure 4 presents the forest plot with 95% confidence intervals across studies.

#### 3.3.6. GRADE Certainty Assessment

**Figure 5:**
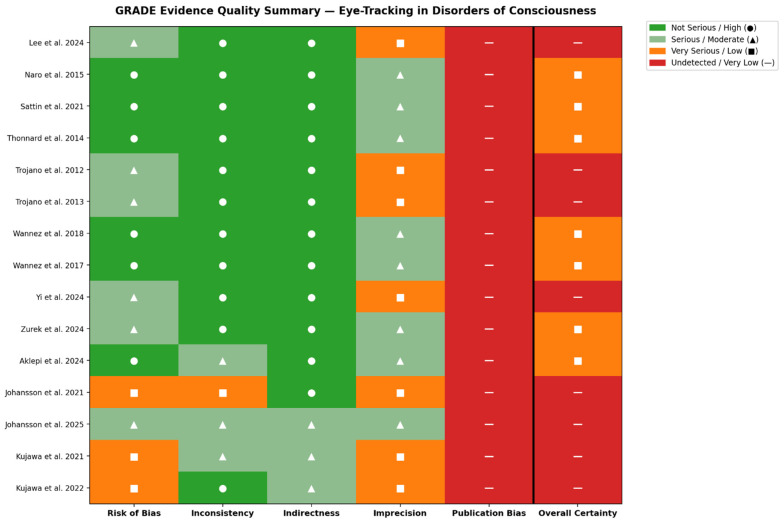
GRADE Evidence Summary. Domain-by domain heatmap across five GRADE domains (risk of bias, inconsistency, indirectness, imprecision, publication bias) and overall certainty for each outcome and article [1,14,15,23,25,27,28,29,30,31,32,33,34,35,36]. Colour-coded heatmap (🟢 Not Serious/🟡 Serious/🟠 Very Serious/🔴 Undetected/Very Low) with the Overall Certainty column separated by a bold vertical line.

The GRADE framework was applied to five key outcomes. Figure 5 presents the GRADE evidence summary and domain-by-domain heatmap. Table 3 presents the Summary of Findings table. The overall certainty of evidence was Low for detection rates and diagnostic discrimination, and Very Low for sensitivity, specificity, and prognostic outcomes. Key reasons for downgrading included: (1) serious risk of bias (blinding universally absent; small samples in most studies); (2) serious inconsistency in detection rates (range 13–100%); (3) very serious imprecision for sensitivity and specificity outcomes (only 4 studies, n ≤ 21 patients with DOC each); and (4) suspected publication bias. The detection rate outcome received a one-level upgrade for large effect size (2.5× relative increase vs. CRS-R). Full domain-by-domain justification is provided in Supplementary Table S2.

## 4. Discussion

This systematic review identified fifteen studies examining eye-tracking technology for consciousness assessment in patients with DOC. The evidence consistently demonstrates that eye-tracking provides objective, quantitative measurement of visual behaviours that complement and augment standard clinical assessment. Five principal themes emerged: (1) enhanced detection of VP and VF compared to clinical observation; (2) critical influence of stimulus type on detection rates; (3) quantitative advantages of eye-tracking over categorical clinical scales; (4) promising diagnostic and prognostic utility of advanced VR and BCI technologies; and (5) preliminary but encouraging prognostic evidence.

The included studies demonstrated considerable heterogeneity across multiple dimensions. Eye tracking technologies ranged from stationary infrared systems [27,28] to wearable devices [1,31], VR-integrated systems [14,25], and hybrid BCI approaches. These systems vary in sampling rate (30–60 Hz), spatial accuracy (0.5–2° visual angle), calibration requirements, and susceptibility to artefacts. The lack of standardisation makes it difficult to determine whether differences in findings reflect true patient characteristics or technical factors.

The enhanced sensitivity of eye-tracking compared to clinical observation suggests that integration of this technology into routine assessment protocols could reduce the unacceptably high misdiagnosis rate in DOC [1,6]. Given that misdiagnosis has profound implications for treatment decisions, prognostic counselling, and ethical considerations, any technology that improves diagnostic accuracy warrants serious consideration. The finding that mirror stimuli achieve 97% detection rates has immediate practical implications for clinical assessment protocols. Based on the GRADE certainty assessment (Low to Very Low), the current evidence supports a conditional (weak) recommendation for eye-tracking as a supplementary tool alongside CRS-R, rather than a strong recommendation for routine replacement. This aligns with existing clinical guidance recommending multimodal assessment.

The detection of visual responses by eye-tracking that are not observed clinically provides empirical support for the concept of “covert consciousness”—the dissociation between preserved awareness and the ability to demonstrate that awareness through voluntary motor responses. This dissociation has important theoretical implications for understanding the neural basis of consciousness and the relationship between awareness and action.

Several limitations affect this study and possible clinical translation of eye-tracking in DOC, as demonstrated by the risk of bias and the certainty assessment. First, the majority of studies included small samples (range n = 6–46), with only two studies exceeding 40 participants [15,29]. Small samples limit statistical power, increase risk of Type II errors, and reduce generalisability. Moreover, blinding of assessors was universally absent or unreported, introducing potential performance and detection bias. Only studies using fully automated algorithms [14,25] partially mitigate this concern. Key confounders, including time since injury, aetiology, medication effects, arousal fluctuations, and sensory impairments, were inconsistently addressed. Finally, while most studies used CRS-R, one used GCS and others used neurophysiological measures or clinical observation without formal scale administration, demonstrating a reference standard variability that deeply affects translatability of this tool. The substantial clinical and methodological heterogeneity precluded quantitative synthesis; all findings are based on narrative synthesis. A further limitation of the included studies, and of eye-tracking technology in the context of DOC assessment more broadly, concerns age-related performance variability. Corneal-reflection-based systems may be affected by corrective lenses, while observational face-tracking systems are frequently trained on databases biased towards younger individuals (approximately 20–45 years), with sparse representation of individuals aged >65 years, which may result in reduced calibration accuracy, increased tracking loss, and higher error rates in older patients with DOC [37,38]. Age-related morphological changes—including orbital bone resorption, repositioning of facial fat pads, and periorbital tissue changes—alter the facial landmarks on which many tracking algorithms rely. The studies reported a mean patient age that varied considerably, with the youngest participant being 7 years old [28] and studies generally including adult population up to 76 years of age. Future studies should explicitly assess ET system performance across age strata and report calibration success rates by age group.

As for this study’s limitations, (a) this review was not prospectively registered in PROSPERO, which is acknowledged as a limitation, even if eligibility criteria were pre-specified to minimise post hoc modification. (b) The search was restricted to English-language publications, which may have excluded relevant studies published in other languages. (c) Full-text data were unavailable for two included studies, with data extracted from abstracts only; this may have introduced underreporting of methodological and statistical details for those studies.

To conclude, eye-tracking technology provides objective, sensitive assessment of visual behaviours in patients with DOC and demonstrates consistent advantages over standard clinical observation. The evidence supports a conditional recommendation for eye-tracking as a supplementary assessment tool alongside CRS-R, with mirror and personally meaningful stimuli recommended to optimise detection rates. Advanced VR-based systems and hybrid BCI approaches show promise for both diagnostic and prognostic applications. However, the Low to Very Low GRADE certainty of the current evidence base underscores the need for rigorous, adequately powered research.

## Figures and Tables

**Table 1:** Characteristics of Included Studies.

First Author & Year	Sample Size	Age Range (Years)	Study Aim	Sample Characteristics	Study Design	Eye-Tracking Protocol	Main Findings
Trojano et al., 2012 [27]	18 (9 VS/UWS, 9 MCS)	19–69	Quantitative assessment of visual behaviour in DOC	Chronic DOC patients; infrared eye-tracking	Observational cross-sectional	Infrared eye tracker; stimuli: moving red circle, moving parrot image, moving photo of relative; VP defined as series of fixations on target	MCS patients showed significantly higher on-target fixations (67.8%) vs. VS/UWS patients (32.2%); quantitative eye-tracking differentiated diagnostic groups
Trojano et al., 2013 [28]	26 (13 VS/UWS, 13 MCS)	VS patients: 21–65; MCS patients: 7–76	Investigate affective saliency effects on visual tracking	Chronic DOC patients	Observational cross-sectional	Infrared eye tracker; stimuli: moving circle, moving parrot, moving photo of close relative	MCS patients showed significantly higher tracking of relative’s photo (37.3%) vs. parrot (29.9%) vs. circle (30.6%); affective saliency modulates visual tracking in MCS but not VS/UWS
Thonnard et al., 2014 [29]	88 (47 MCS−, 41 MCS+)	Mean 51 ± 20	Detection of visual pursuit in MCS; effects of stimuli and visual plane	MCS patients assessed with CRS-R	Observational cross-sectional	CRS-R protocol; stimuli: mirror, person, object; horizontal and vertical planes assessed	97% tracked mirror, 69% tracked person, 57% tracked object; mirror most sensitive stimulus; more horizontal tracking overall; MCS− showed more horizontal preference than MCS+
Naro et al., 2015 [30]	25 DOC, 2 EMCS, 1 LIS and 20 healthy controls	Healthy controls: Mean 56.5 ± 6.3; DOC subgroups: Mean 52–57	Visual fixation in chronic DOC for differential diagnosis	Chronic DOC patients	Observational cross-sectional	Eye-tracking assessment of visual fixation	Visual fixation patterns differentiate diagnostic categories in chronic DOC; supports use of eye-tracking for differential diagnosis
Wannez et al., 2017 [31]	123 DOC	Mean 40 ± 14; Range 18–72	Assess mirror efficiency in visual pursuit assessment in MCS	MCS patients	Observational cross-sectional	Eye-tracking with mirror stimulus; comparison with other stimuli	Mirror confirmed as most efficient stimulus for detecting visual pursuit in MCS patients; supports preferential use of mirror in assessment protocols
Wannez et al., 2018 [32]	31 DOC and 23 healthy controls	DOC patients: Mean 40.23 ± 13.19; Healthy subjects: Mean 28 ± 7	Objective assessment of visual pursuit in DOC using wearable eye tracker	MCS patients	Exploratory observational	Wearable eye tracker; assessment of visual pursuit	Wearable eye-tracking detected visual pursuit responses not observed through clinical assessment alone; objective quantification possible
Johansson et al., 2021 [23]	4 DOC	22–43	Methodological aspects of wearable eye-tracker for DOC assessment	DOC patients	Methodological study	Wearable eye tracker; evaluation of technical and practical aspects	Wearable eye-trackers are feasible for DOC assessment; technical considerations include calibration challenges and need for standardised protocols
Kujawa et al., 2021 [33]	6 (all VS/UWS)	18–62	Assessment of language functions in DOC using eye-tracking AAC	6 VS/UWS patients (age 18–62; 3 men, 3 women); aetiology: sudden circulatory arrest; 4–6 months post-injury; diagnosed with GCS ≤ 8	Longitudinal case series (1, 6, 12 months)	C-Eye Pro infrared system; tasks: selecting pictograms, yes/no questions, sentence matching; 60 min sessions with 25 language tasks; calibration before each session	Average 70.45% task completion rate; high performance suggests potential misdiagnosis (patients may be MCS rather than VS/UWS); high intra-individual variability noted
Sattin et al., 2021 [34]	58 DOC	Mean 66.72 ± 25.07	Visual fixation in DOC: predictive models for differential diagnosis	DOC patients	Observational cross-sectional	Eye-tracking assessment of visual fixation; development of predictive models	Visual fixation patterns can be modelled to predict diagnostic category; supports integration of eye-tracking into diagnostic algorithms
Kujawa et al., 2022 [35]	12 DOC	26–67	Monitoring eye movements by visual stimulus type in impaired consciousness	Patients with impaired consciousness	Observational cross-sectional	Eye-tracking with multiple stimulus types; comparison of responses	Stimulus type significantly influences eye movement patterns and detection rates; supports systematic stimulus selection in protocols
Aklepi et al., 2024 [25]	10 DOC and 20 healthy controls	Mean 29 ± 17	Covert tracking to immersive stimuli in TBI with DOC	TBI patients with DOC	Observational cross-sectional	Immersive stimuli presentation; assessment of covert tracking	Immersive stimuli can elicit covert tracking responses in TBI patients with DOC; may reveal consciousness signs not detected with standard stimuli
Lee et al., 2024 [14]	21 (15 DOC, 6 healthy controls)	DOC patients: Median 67 (IQR: 64.5–72.5); Healthy controls: Median 55 (IQR: 52.3–58.3)	VR-based eye-tracking system for impaired consciousness; validation of RPTL biomarker	DOC group: median age 67 (IQR 64.5–72.5), 60% women; etiologies: SAH, SDH, ICH, HBI, TBI; chronicity 32–560 days; CRS-R assessment	Observational cross-sectional with 1-year follow-up	HTC Vive HMD with infrared pupil tracking (60 Hz); 9 visuoauditory VR stimuli; RPTL-V and RPTL-A calculated (pupil trajectory during stimuli vs. blank); validated against VEP/BAEP	RPTL-V cut-off 14.737 (sensitivity 100%, specificity 88.9%); RPTL-A cut-off 30.019 (sensitivity 88.9%, specificity 83.3%); overt tracking predicted command-following at 1 year in 62.5%; one patient showed RPTL response not detected by CRS-R
Yi et al., 2024 [36]	9 DOC, 1 LIS, 10 healthy controls	Patients: Mean 38.8 ± 22.2; Healthy volunteers: Mean 24.0 ± 1.2	Hybrid BCI integrating EEG and eye-tracking for DOC	DOC patients	Observational cross-sectional	Hybrid BCI system combining EEG and eye-tracking; multimodal assessment	Integration of EEG and eye-tracking provides complementary information for consciousness assessment; hybrid approach may improve diagnostic accuracy
Zurek et al., 2024 [15]	46 DOC	≥18 (specific range not provided)	Can eye-tracking help assess consciousness in non-verbal brain injury patients; MCSD test vs. CRS-R	Non-verbal brain injury patients; multi-centre trial, Poland 2022–2023	Multi-centre clinical trial	MCSD (Multimodal Consciousness State Detection) test incorporating eye-tracking; comparison with CRS-R	Eye-tracking-based MCSD test provides additional diagnostic information beyond CRS-R; supports integration into clinical assessment protocols
Johansson et al., 2025 [1]	12 (outpatients with prolonged DOC)	Median 43; Range 18–64	Eye-tracking to support assessment of prolonged DOC	12 outpatients with prolonged DOC	Observational cross-sectional	Wearable eye tracker; comparison with clinical assessment	Eye-tracking detected responses in 46.2% of trials vs. 18.1% by clinical assessment alone; demonstrates superior sensitivity of objective eye-tracking

Systematic Review: Eye-Tracking Technology for Assessing Disorders of Consciousness—A PRISMA 2020-Compliant Systematic Review. Note: DOC = Disorders of Consciousness; VS/UWS = Vegetative State/Unresponsive Wakefulness Syndrome; MCS = Minimally Conscious State; CRS-R = Coma Recovery Scale-Revised; VP = Visual Pursuit; TBI = Traumatic Brain Injury; BCI = Brain–Computer Interface; VR = Virtual Reality; RPTL = Relative Pupil Trajectory Length; EEG = Electroencephalography; GCS = Glasgow Coma Scale; HMD = Head-Mounted Display; SAH = Subarachnoid Haemorrhage; SDH = Subdural Haematoma; ICH = Intracerebral Haemorrhage; HBI = Hypoxic Brain Injury; IQR = Interquartile Range.

**Table 2:** Risk of Bias Assessment for Included Studies.

Study	Study Design	Selection Bias	Performance Bias	Detection Bias	Attrition Bias	Reporting Bias	Other Bias	Overall Risk
Lee et al., 2024 [14]	Observational prospective cohort (single-centre, n = 15)	Unclear (inclusion/exclusion criteria specified but sampling method not clearly described)	Unclear (VR protocol described but uncalibrated pupil trajectories acknowledged; standardisation not fully detailed)	Low (VEP/BAEP assessed by two domain experts via visual inspection; objective electrophysiological measures; ROC-determined cut-offs)	High (1/3 covert tracking patients died before follow-up; follow-up methods varied face-to-face vs. video calls; missing data not fully quantified)	Unclear (no pre-registration mentioned; sample size limitation acknowledged; one discordance case noted)	Low (no competing interests declared; age discrepancy between DOC and controls noted but deemed negligible)	High
Naro et al., 2015 [30]	Interventional prospective sham-controlled crossover (single-centre, n = 25)	Low (inclusion/exclusion criteria clearly defined; consecutive enrollment from neurorehabilitation unit)	Low (standardised TMS/tDCS protocols; sham-controlled; randomised order; healthy controls and examiners blinded)	Low (blinded examiners analysing EEG data; objective neurophysiological measures; standardised analysis procedures)	Unclear (no explicit dropout or missing data reported; all 25 patients appear to have completed protocol)	Low (methods and outcomes clearly described; limitations acknowledged; no pre-registration but systematic protocol)	Low (no conflicts of interest declared; funded by Italian Ministry of Health)	Low
Sattin et al., 2021 [34]	Observational prospective cohort (single-centre, n = 58)	Low (consecutive enrollment; clear inclusion/exclusion criteria; representative sample from neurorehabilitation unit)	Low (standardised CRS-R and VEP protocols; trained examiners; objective electrophysiological measures)	Low (objective VEP measurements; blinded analysis not stated but objective measures reduce bias; ROC analysis for cut-offs)	Low (all 58 patients completed assessments; no missing data reported)	Low (comprehensive reporting of methods and outcomes; limitations discussed; statistical methods appropriate)	Low (no conflicts of interest declared; funded by Italian Ministry of Health)	Low
Thonnard et al., 2014 [29]	Observational prospective cohort (single-centre, n = 88 MCS)	Low (consecutive enrollment; clear diagnostic criteria; representative MCS sample)	Low (standardised CRS-R protocol; trained examiners; systematic assessment of pursuit across multiple stimuli)	Unclear (clinical observation without objective measurement; no blinding mentioned; subjective judgement of pursuit)	Low (all 88 patients completed assessments; comprehensive data reported)	Low (detailed reporting of pursuit patterns; limitations discussed; no pre-registration but systematic protocol)	Low (no conflicts of interest apparent; academic study)	Unclear
Trojano et al., 2012 [27]	Observational case–control (single-centre, n = 18 DOC: 9 MCS, 9 UWS)	Unclear (convenience sample; small sample size; no details on consecutive enrollment or sampling method)	Low (standardised infrared eye-tracking protocol using MyTobii T60; objective measurements; fixed stimulus presentation)	Low (objective eye-tracking measurements; automated data recording; chi-square analysis for statistical comparison)	Low (all 18 patients completed protocol; no missing data reported)	Unclear (no pre-registration; small sample acknowledged; unclear if all planned analyses reported)	Low (no conflicts of interest apparent; academic study)	Unclear
Trojano et al., 2013 [28]	Observational case series (single-centre, sample size unclear)	High (sample size not clearly reported; convenience sample; unclear enrollment procedures)	Low (standardised infrared eye-tracking protocol using MyTobii T60; objective measurements; controlled stimulus presentation)	Low (objective eye-tracking measurements; automated data recording; chi-square analysis)	Unclear (sample size unclear; no attrition data reported)	High (sample size not clearly stated; limited methodological details; unclear if all outcomes reported)	Low (no conflicts of interest apparent; academic study)	High
Wannez et al., 2018 [32]	Observational prospective cohort (single-centre, n = 31 patients, 23 controls)	Low (clear inclusion/exclusion criteria; consecutive enrollment; representative sample)	Low (standardised automated device protocol; C-score and M-score algorithms; objective measurements)	Low (automated scoring algorithms; objective measurements; blinded validation against CRS-R; 100% subject-level agreement κ = 1.0)	Low (all 54 participants completed protocol; comprehensive data reported)	Low (detailed reporting of methods and validation; limitations discussed; systematic protocol)	Low (no conflicts of interest declared; funded by Belgian National Funds for Scientific Research)	Low
Wannez et al., 2017 [31]	Observational prospective cohort (single-centre, n = 123 DOC)	Low (consecutive enrollment; clear diagnostic criteria; large representative sample)	Low (standardised CRS-R protocol; trained examiners; repeated assessments up to 5 times)	Low (standardised CRS-R with established reliability; multiple assessments reduce bias; systematic protocol)	Low (all 123 patients completed multiple assessments; comprehensive data reported)	Low (detailed reporting of misdiagnosis rates; limitations discussed; systematic protocol)	Low (no conflicts of interest declared; funded by Belgian National Funds for Scientific Research)	Low
Yi et al., 2024 [36]	Observational case–control (single-centre, n = 10 patients, 10 controls)	Unclear (convenience sample; small sample size; no details on consecutive enrollment)	Low (standardised hybrid BCI protocol combining EEG and eye-tracking; objective measurements; controlled stimulus presentation)	Low (objective EEG and eye-tracking measurements; automated data recording; statistical analysis of communication accuracy)	Low (all 20 participants completed protocol; no missing data reported)	Unclear (no pre-registration; small sample acknowledged; unclear if all planned analyses reported)	Low (funded by National Natural Science Foundation of China; no conflicts of interest declared)	Unclear
Zurek et al., 2024 [15]	Observational prospective cohort (single-centre, n = 46 analysed from 66 recruited)	Unclear (30% excluded; inclusion/exclusion criteria specified but sampling method not fully described)	Low (standardised C-EYE X eye-tracker protocol; MCSD algorithm; objective measurements; controlled testing environment)	Low (objective eye-tracking measurements; automated MCSD scoring; blinded comparison with CRS-R)	High (30% excluded: 20 patients excluded for various reasons; missing data not fully quantified; no analysis of excluded vs. included)	Unclear (no pre-registration mentioned; sample size limitation acknowledged; need for larger studies noted)	Low (funded by government grant and company; authors declare scientific rigour; no competing interests)	High
Aklepi et al., 2024 [25]	Observational prospective pilot (single-centre, n = 10 TBI)	Low (single-centre convenience sample but clear inclusion/exclusion criteria; recruited 20 healthy volunteers and 10 TBI subjects)	Low (standardised VET protocol using Tobii Pro Glasses 2; predefined tracking criteria; screen position confirmed; calibration attempted)	Low (multiple levels of blinding implemented; objective eye-tracking measurements 50 Hz; two independent examiners Cohen’s κ = 0.8 CI 0.7–0.96)	Low (84 tested trials; one subject and one trial did not generate sufficient ML data resulting in 72 tested trials; follow-up: all accounted at 6 months, 1 lost/1 deceased/1 regressed at 12 months)	Low (pre-registered ClinicalTrials.gov NCT04712591; analysis plan pre-determined; acknowledged as pilot not powered for differences)	Low (multiple NIH and institutional funding; one author minority shareholder at iCE Neurosystems; no industry sponsorship of this study)	Low
Johansson et al., 2021 [23]	Observational methodological case series (single-centre, n = 4)	High (four patients from single rehabilitation clinic; convenience sample; no randomisation; very small sample)	Unclear (CRS-R administered per standard protocol; Tobii Pro Glasses 2 used 50 Hz; arousal optimisation attempted; standardisation not fully detailed)	Low (blinded scoring of clinical vs. eye-tracking protocols; objective eye-tracking measurements; stepwise analysis)	High (Patient 3 had complete data for only 21% of trials due to gaze deviation; data retrieved 96–100% for 3/4 patients but only 21% for one; no discussion of missing data impact)	Unclear (no pre-registration; feasibility study with exploratory aims; unclear if all planned outcomes reported)	Low (funded by SLL Innovation and Promobilia; no conflicts of interest apparent; no industry sponsorship)	High
Johansson et al., 2025 [1]	Observational case series (single-centre, n = 12)	High (convenience sample recruited consecutively from single rehabilitation unit; small sample; only prolonged PDOC patients; limits generalisability)	Unclear to High (standardised CRS-R protocol; equipment comfort issues noted: 4 patients showed poor responses wearing eye-tracker but improved without it; calibration problems and difficulties linking eye movements to stimuli)	Low (blinded scoring by different assessors; objective eye-tracking measurements; however recorded eye movements often subtle, strained, frequently interrupted)	Unclear (response data obtained from 238/288 trials 82.6%; no data for 50 trials due to closed eyes or extreme angles; eye-tracking data obtained median 89.6% range 37.5–100%; unclear if patient-level attrition)	Unclear (no pre-registration; unclear if all planned outcomes reported)	Low (funded by Innovationsfonden Region Stockholm and Promobilia; no conflicts of interest declared)	High
Kujawa et al., 2021 [33]	Observational longitudinal (1, 6, 12 months; single-centre, n = 6)	High (six patients aged 18–62; convenience sample; all UWS from sudden circulatory arrest; small sample; no randomisation)	High (calibration procedure described; 25 tasks per session randomly selected; session frequency varied due to patient health and willingness; lack of standardisation in session frequency)	Unclear (no blinding reported; objective eye-tracking measurements; monitored eye opening and gaze direction; unclear who scored outcomes and whether blinded)	Unclear (variable cooperation noted in Patients 3, 5, 6: sometimes considerable decrease in efficiency or no cooperation; no explicit dropout rates or missing data handling; frequency varied due to changing health)	Unclear (no pre-registration; unclear if all outcomes reported; limited statistical analysis)	Low (no conflicts of interest declared; no industry sponsorship apparent)	High
Kujawa et al., 2022 [35]	Observational examining eyeball movement patterns (single-centre, n = 12 final from 20 initial)	High (recruited from single Palliative Care Centre; started N = 20, final N = 12 after exclusions and deaths; convenience sample; small sample acknowledged as limitation)	Low to Unclear (standardised protocol using C-Eye device 30 Hz, 0.4° accuracy, 40 cm/s velocity threshold; one-point calibration; fixed distance 50 cm; monitor position adjusted; no head stabilisation)	Low (automated data recording and analysis; assessment independent of human investigator; objective measurement of fixations and saccades; built-in software detected all eye movements automatically)	Very Serious (3 failed calibrations excluded; 5 persons died during project; started N = 20, final N = 12: 40% attrition; no analysis of differences between completers and non-completers)	Unclear (no pre-registration; unclear if all planned outcomes reported)	Low (no conflicts of interest declared)	High

**Table 3:** GRADE Summary of Findings.

Study	No. of Participants	Risk of Bias	Inconsistency	Indirectness	Imprecision	Publication Bias	Overall Certainty
Lee et al., 2024 [14]	15 DOC patients	Serious (high attrition bias; unclear selection and performance bias; varied follow-up methods)	Not Serious (single study; internal consistency not assessable)	Not Serious (direct comparison of eye-tracking RPTL vs. CRS-R; relevant patient population; 1-year functional outcome)	Very Serious (very small sample n = 15; only 1 patient showed covert tracking without CRS-R response; wide variation in follow-up assessment methods)	Undetected (single study; no competing interests declared)	Very Low
Naro et al., 2015 [30]	25 DOC patients (15 UWS, 10 MCS)	Not Serious (low risk across all domains; blinded sham-controlled crossover design; standardised protocols)	Not Serious (single study; consistent findings across cortical connectivity measures)	Not Serious (directly addresses cortical connectivity in DOC; relevant population; appropriate intervention and comparator)	Serious (moderate sample n = 25; no confidence intervals reported for some measures; single-centre study)	Undetected (single study; no conflicts of interest; funded by Italian Ministry of Health)	Moderate
Sattin et al., 2021 [34]	58 DOC patients	Not Serious (low risk across all domains; consecutive enrollment; standardised protocols; objective measures)	Not Serious (single study; consistent VEP findings across patient groups)	Not Serious (directly addresses VEP as predictor of consciousness recovery; relevant population; appropriate outcome measures)	Not Serious (adequate sample n = 58; confidence intervals reported for ROC analysis; AUC 0.832 CI 0.724–0.940)	Undetected (single study; no conflicts of interest; funded by Italian Ministry of Health)	High
Thonnard et al., 2014 [29]	88 MCS patients	Not Serious to Serious (low risk overall but unclear detection bias due to subjective clinical observation without objective measurement)	Not Serious (single study; consistent pursuit patterns across stimuli types)	Not Serious (directly addresses visual pursuit patterns in MCS; relevant population; clinically relevant stimuli)	Not Serious (adequate sample n = 88; comprehensive data on pursuit patterns across multiple stimuli)	Undetected (single study; no conflicts of interest apparent)	Moderate
Trojano et al., 2012 [27]	18 DOC (9 MCS, 9 UWS)	Serious (unclear selection bias; small sample; convenience sampling; unclear reporting bias)	Not Serious (single study; consistent findings: MCS 78% above chance, UWS 0% above chance)	Not Serious (directly addresses eye-tracking detection in DOC; relevant population; objective measurements)	Serious (small sample n = 18; no confidence intervals reported; chi-square *p* < 0.001 but limited statistical detail)	Undetected (single study; no conflicts of interest apparent)	Low
Trojano et al., 2013 [28]	Sample size unclear	Very Serious (high selection bias: sample size not clearly reported; high reporting bias: limited methodological details; unclear attrition)	Not Serious (single study; consistent finding: face vs. circle χ^2^ = 43.40 *p* < 0.001)	Not Serious (directly addresses emotional stimulus processing via eye-tracking; relevant population)	Very Serious (sample size unclear; no confidence intervals; limited statistical reporting)	Undetected (single study; no conflicts of interest apparent)	Very Low
Wannez et al., 2018 [32]	31 patients, 23 controls (total 54)	Not Serious (low risk across all domains; automated scoring; blinded validation; 100% subject-level agreement κ = 1.0)	Not Serious (single study; consistent validation results across subgroups; M-score sensitivity/specificity 80–100%)	Not Serious (directly addresses automated eye-tracking device validation; relevant population; appropriate comparator CRS-R)	Not Serious (adequate sample n = 54; confidence intervals not reported but perfect agreement κ = 1.0; sensitivity/specificity 80–100% across subgroups)	Undetected (single study; no conflicts of interest; funded by Belgian National Funds)	High
Wannez et al., 2017 [31]	123 DOC patients	Not Serious (low risk across all domains; consecutive enrollment; large sample; standardised repeated CRS-R assessments)	Not Serious (single study; consistent finding: 5 assessments needed to reduce misdiagnosis to 5%)	Not Serious (directly addresses misdiagnosis rates in DOC; relevant population; clinically important outcome)	Not Serious (large sample n = 123; precise estimates of misdiagnosis rates; visual pursuit in 2/6 missed MCS-, fixation in 1/6)	Undetected (single study; no conflicts of interest; funded by Belgian National Funds)	High
Yi et al., 2024 [36]	10 patients, 10 controls (total 20)	Serious (unclear selection and reporting bias; small sample; convenience sampling)	Not Serious (single study; consistent finding: 5/10 patients 50% achieved significant communication mean 76.1 ± 7.9%)	Not Serious (directly addresses hybrid BCI communication in DOC; relevant population; P5 and P10 communicated but CRS-R communication = 0)	Serious (small sample n = 20; no confidence intervals reported; limited statistical detail)	Undetected (single study; no conflicts of interest; funded by National Natural Science Foundation of China)	Low
Zurek et al., 2024 [15]	46 analysed (from 66 recruited)	Serious (high attrition bias: 30% excluded; unclear selection and reporting bias; missing data not fully quantified)	Not Serious (single study; consistent correlations across all time points T1–T5; consistent with adaptation effect: T1–T2 familiarisation, T3+ reliable)	Not Serious (directly addresses eye-tracking technology validation C-EYE X MCSD; relevant patient population DOC requiring neurorehabilitation; appropriate comparator CRS-R)	Serious (moderate sample n = 46 analysed from 66 recruited; wide confidence intervals for some regression coefficients, e.g., T1 β_0_: −14.58 to 19.48; acknowledged need for larger studies)	Undetected (single study; funded by government grant and company; authors declare scientific rigour)	Low
Aklepi et al., 2024 [25]	10 TBI patients, 20 healthy volunteers	Not Serious (multiple levels of blinding; pre-registered; standardised protocols; low risk across all domains)	Serious (single-centre study with no replication; pilot study not powered for efficacy; machine learning algorithm showed only moderate agreement with human examiners Cohen’s κ = 0.5 CI 0.3–0.7)	Not Serious (direct measurement of target outcome tracking ability; relevant population unresponsive TBI patients; immersive video stimuli directly relevant to clinical assessment)	Serious (small sample n = 10 TBI subjects; wide confidence intervals on some measures, e.g., Cohen’s κ for ML: 0.3–0.7; pilot study acknowledged as not powered for differences; follow-up limited by attrition: 1 lost, 1 deceased, 1 regressed at 12 months)	Undetected (pre-registered study; no evidence of selective reporting)	Low
Johansson et al., 2021 [23]	4 patients	Very Serious (very small sample n = 4; high attrition for one patient 79% data loss; no randomisation; convenience sampling; unclear reporting of outcomes)	Very Serious (high variability in agreement between patients 54–80%; one patient with extreme data loss; variable performance within subjects and test items)	Not Serious (direct assessment of target outcome eye movements during CRS-R; relevant population PDOC patients)	Very Serious (extremely small sample n = 4; no confidence intervals reported; wide variation in results between patients; 75% data loss in one patient)	Undetected (feasibility study with negative/mixed results published; no evidence of selective reporting)	Very Low
Johansson et al., 2025 [1]	12 patients	Serious (small convenience sample n = 12; equipment comfort issues affected 4 patients; methodological challenges with subtle eye movements and calibration)	Serious (poor inter-method agreement Cohen’s κ = 0.073 CI −0.031 to 0.177; wide range in data retrieval rates 37.5–100%; variable results across test items: some significant, some not)	Serious (only prolonged PDOC patients included; equipment design limitations may have affected results; generalisability limited)	Serious (small sample n = 12; wide confidence interval on Cohen’s κ: −0.031 to 0.177; 50/288 trials 17.4% had no data)	Undetected (no evidence of selective reporting; limitations openly discussed)	Very Low
Kujawa et al., 2021 [33]	6 UWS patients	Very Serious (very small sample n = 6; no blinding reported; variable session frequency and patient cooperation; convenience sample)	Serious (high variability in individual patient performance SD range 9.1 to 33.4; three patients showed considerable variability and sometimes no cooperation; non-significant results but trends observed)	Serious (study claims patients diagnosed as UWS but performed tasks suggesting MCS; diagnostic accuracy questioned by authors themselves; unclear clinical relevance of task completion percentages)	Very Serious (extremely small sample n = 6; no confidence intervals reported; wide standard deviations; non-significant statistical tests: *p* < 0.713 and *p* < 0.401)	Undetected (no evidence of selective reporting; negative results non-significance reported)	Very Low
Kujawa et al., 2022 [35]	12 final (from 20 initial)	Very Serious (small final sample n = 12; very high attrition rate 40%: 3 failed calibrations, 5 died; convenience sample from single centre; no analysis of attrition impact)	Not Serious (consistent finding across all patients: dynamic > static; all statistical comparisons significant in same direction: *p* = 0.028, *p* = 0.002, *p* = 0.030)	Unclear (relevant population DOC patients; direct measurement of eye movements; however clinical relevance of static vs. dynamic distinction unclear; study limitation acknowledged: small sample and high mortality)	Very Serious (very small final sample n = 12; started n = 20, lost 40%; no confidence intervals reported; limited generalisability acknowledged by authors)	Undetected (no evidence of selective reporting; limitations openly discussed)	Very Low

## Data Availability

Data extraction tables, risk of bias assessments, and visualisation scripts are available upon request from the corresponding author. No new data were created or analysed in this study.

## Supplementary Materials

The following supporting information can be downloaded at: https://www.mdpi.com/article/10.3390/brainsci16060590/s1, Table S1: PRISMA 2020 Checklist; File S1: Complete Boolean search string; Table S2: GRADE profile summary.
