# Supplementary material for: Eye-Tracking Assessment in Patients with Disorders of Consciousness: A Systematic Review
Source: Brain Sci. 2026 May 30;16(6):590. doi: 10.3390/brainsci16060590 (PMC13297577; doi:10.3390/brainsci16060590)
Supplement: Supplementary file 1 [file brainsci-16-00590-s001.zip › Supplemenaty Table S2.pdf]

Supplementary File 3 — GRADE Evidence Profile

Part 1: Summary of Findings Table

Outcome	Number of Studies	Number of Patients	Effect Estimate	Certainty of Evidence (GRADE)	Interpretation
1. Detection Rate (ET vs. CRS-R)	**n = 15 [Study count revised from 16 to 15 following removal of Trojano et al. (2018), a review article that did not meet eligibility criteria. GRADE certainty ratings have been reconsidered and remain unchanged.]**	6–88 per study	ET detected visual responses in 46.2% vs. CRS-R 18.1% (Johansson et al., 2025); 2.5-fold increase	⨁⨁○○ LOW	Eye-tracking detects significantly more visual responses than clinical observation alone
2. Diagnostic Discriminatio	**n = 15 [Study count**	6–88 per study	Mirror: 97% detection; Person: 69%;	⨁⨁○○ LOW	Mirror stimuli achieve highest

n (Stimulus Type)	**revised from 16 to 15 following removal of Trojano et al. (2018), a review article that did not meet eligibility criteria. GRADE certainty ratings have been reconsidered and remain unchanged.]**		Object: 57% (Trojano et al., 2012)		detection rates; stimulus hierarchy established
3. Affective Salience Response	**n = 15 [Study count revised from 16 to 15 following removal of Trojano et al. (2018), a review article that did not meet eligibility criteria. GRADE certainty ratings have been reconsidered and remain**	6–88 per study	MCS patients: 37.3% affective vs. 29.9–30.6% neutral stimuli	⊕⊕○○ LOW	Affectively salient stimuli elicit stronger tracking responses in MCS patients

	**unchanged.]**				
4. Sensitivity and Specificity (VR-based ET)	**n = 15 [Study count revised from 16 to 15 following removal of Trojano et al. (2018), a review article that did not meet eligibility criteria. GRADE certainty ratings have been reconsidered and remain unchanged.]**	88 patients	Sensitivity: 100% (95% CI: 88.9–100%); Specificity: 88.9% (95% CI: 70.8–97.6%) (Lee et al., 2024) **[PREPRINT — ResearchSquare, not peer-reviewed. The sole evidence source for this outcome is a non-peer-reviewed preprint. This is reflected in the Very Low certainty rating and should be reconsidered upon formal publication.]**	⨁○○○ VERY LOW	High diagnostic accuracy for VR-based RPTL-V biomarker, but based on single preprint study
5. Prognostic Value	**n = 15 [Study count revised from 16 to 15 following removal of Trojano et al. (2018), a review article that did not meet eligibility criteria. GRADE certainty ratings**	8 patients with overt tracking	62.5% (5/8) of overt trackers demonstrated command-following at 1-year follow-up (Johansson et al., 2025)	⨁○○○ VERY LOW	Preliminary evidence for prognostic value; very small sample limits confidence

	**have been reconsidered and remain unchanged.]**				

GRADE Certainty Levels:

• ⨁⨁⨁⨁ HIGH: Very confident that the true effect lies close to the estimate

• ⨁⨁⨁○ MODERATE: Moderately confident; true effect is likely close to the estimate but may be substantially different

• ⨁⨁○○ LOW: Limited confidence; true effect may be substantially different from the estimate

• ⨁○○○ VERY LOW: Very little confidence; true effect is likely substantially different from the estimate

---

Part 2: Detailed GRADE Assessment by Outcome

Outcome 1: Detection Rate (Eye-Tracking vs. CRS-R)

Question: Does eye-tracking detect visual responses more frequently than CRS-R clinical observation in DOC patients?

Key Evidence: Johansson et al. (2025) reported ET detection in 46.2% vs. CRS-R 18.1% (2.5-fold increase); multiple studies report detection of covert visual responses missed by clinical assessment.

GRADE Domain	Rating	Justification
X1. Risk of Bias	Serious (−1)	All studies lack blinding of assessors; CRS-R and ET assessments not conducted independently; high risk of performance and detection bias. No study used double-blind protocol.
X2. Inconsistency	Not serious (0)	Consistent direction of effect across studies: ET consistently detects more responses than CRS-R alone. Magnitude varies (detection rates 18–97%) due to stimulus and population differences, but directional consistency is strong.
X3. Indirectness	Not serious (0)	Studies directly address the question in target population (prolonged DOC patients) using relevant intervention (ET) and comparator (CRS-R).
X4. Imprecision	Serious (−1)	Small sample sizes (n = 6–88 per study); wide confidence intervals not reported in most studies; single-center designs limit generalizability.
X5. Publication Bias	Suspected (−1)	**Rationale: Publication bias is suspected based on: (1) near-absence of null results in the included set — all included studies report positive ET performance metrics; (2) selective outcome reporting risk in small DOC studies where negative findings are less likely to be submitted or accepted; (3) the grey literature search did not identify unpublished studies with null results;**

		**(4) no contact with authors of registered but unpublished trials was feasible. Fewer than 10 studies contribute to this outcome; funnel plot analysis is therefore statistically inappropriate (Sterne et al., 2011). This rating is based on narrative assessment per GRADE guidance.**

Starting Certainty: Observational studies = LOW (⊕⊕○○)

Downgrades: Risk of bias (−1), Imprecision (−1), Publication bias (−1)

Upgrades: None

Final Certainty: ⊕⊕○○ LOW

Outcome 2: Diagnostic Discrimination (Stimulus Type Effects)

Question: Do different stimulus types (mirror, person, object) produce different detection rates in DOC patients?

Key Evidence: Trojano et al. (2012): Mirror 97%, Person 69%, Object 57% detection rates; stimulus hierarchy consistently observed across studies.

GRADE Domain	Rating	Justification
X1. Risk of Bias	Serious (−1)	Lack of blinding; stimulus presentation order not randomized in most

		studies; risk of carryover effects and learning bias.
X2. Inconsistency	Not serious (0)	Consistent stimulus hierarchy across studies: mirror > person > object. Absolute detection rates vary, but relative ordering is consistent.
X3. Indirectness	Not serious (0)	Direct evidence in target population with relevant stimulus comparisons.
X4. Imprecision	Serious (−1)	Small sample sizes; confidence intervals not reported; limited replication of stimulus comparisons.
X5. Publication Bias	Suspected (−1)	**Rationale: Publication bias is suspected based on: (1) near-absence of null results in the included set — all included studies report positive ET performance metrics; (2) selective outcome reporting risk in small DOC studies where negative findings are less likely to be submitted or accepted; (3) the grey literature search did not identify unpublished studies with null results; (4) no contact with authors of registered but unpublished trials was feasible. Fewer than 10 studies contribute to this outcome; funnel plot analysis is therefore statistically inappropriate (Sterne et al., 2011). This rating is based on narrative assessment per GRADE guidance.**

Starting Certainty: Observational studies = LOW (⊕⊕○○)

Downgrades: Risk of bias (−1), Imprecision (−1), Publication bias (−1)

Upgrades: None

Final Certainty: ⊕⊕○○ LOW

Outcome 3: Affective Salience Response

Question: Do affectively salient stimuli (e.g., familiar faces, emotional content) elicit stronger tracking responses than neutral stimuli in DOC patients?

Key Evidence: Multiple studies report stronger tracking to affective vs. neutral stimuli in MCS patients (37.3% vs. 29.9–30.6%); effect less pronounced in VS/UWS.

GRADE Domain	Rating	Justification
X1. Risk of Bias	Serious (−1)	Lack of blinding; subjective classification of "affective" vs. "neutral" stimuli; inconsistent operationalization across studies.
X2. Inconsistency	Not serious (0)	Consistent direction of effect (affective > neutral) in MCS patients across studies; heterogeneity in VS/UWS is expected given diagnostic group differences.
X3. Indirectness	Not serious (0)	Direct evidence in target population with relevant stimulus comparisons.

X4. Imprecision	Serious (−1)	Small sample sizes; wide variability in effect magnitude; confidence intervals not consistently reported.
X5. Publication Bias	Suspected (−1)	**Rationale: Publication bias is suspected based on: (1) near-absence of null results in the included set — all included studies report positive ET performance metrics; (2) selective outcome reporting risk in small DOC studies where negative findings are less likely to be submitted or accepted; (3) the grey literature search did not identify unpublished studies with null results; (4) no contact with authors of registered but unpublished trials was feasible. Fewer than 10 studies contribute to this outcome; funnel plot analysis is therefore statistically inappropriate (Sterne et al., 2011). This rating is based on narrative assessment per GRADE guidance.**

Starting Certainty: Observational studies = LOW (⨁⨁○○)

Downgrades: Risk of bias (−1), Imprecision (−1), Publication bias (−1)

Upgrades: None

Final Certainty: ⨁⨁○○ LOW

Outcome 4: Sensitivity and Specificity (VR-Based Eye-Tracking)

Question: What is the diagnostic accuracy (sensitivity and specificity) of VR-based eye-tracking for detecting consciousness in DOC patients?

Key Evidence: Lee et al. (2024, preprint): Sensitivity 100% (95% CI: 88.9–100%), Specificity 88.9% (95% CI: 70.8–97.6%) for RPTL-V biomarker in 88 patients.

**GRADE Domain**	**Rating**	**Justification**
X1. Risk of Bias	Serious (−1)	Single study; preprint (not peer-reviewed); blinding status unclear; reference standard (CRS-R) applied by same team; risk of incorporation bias.
X2. Inconsistency	Not assessable (0)	Single study; cannot assess consistency.
X3. Indirectness	Not serious (0)	Direct evidence in target population with relevant diagnostic test (VR-based ET) and reference standard (CRS-R).
X4. Imprecision	Serious (−1)	Single study with n = 88; confidence intervals wide for specificity (70.8–97.6%); no replication.
X5. Publication Bias	Unlikely serious (0)	**Rationale: Publication bias is rated as unlikely serious for this outcome because: (1) the single contributing study (Lee et al., 2024, preprint) is a prospective diagnostic accuracy study with pre-specified outcomes; (2) the study reports both sensitivity and specificity, reducing selective metric reporting; (3) however,**

		**note that this study is a non-peer-reviewed preprint, which introduces uncertainty about reporting completeness. This rating may require revision upon peer-reviewed publication.**

Starting Certainty: Observational studies = LOW (⨁⨁○○)

Downgrades: Risk of bias (−1), Imprecision (−1)

Upgrades: None

Final Certainty: ⨁○○○ VERY LOW

Note: This outcome is based entirely on a single preprint study (Lee et al., 2024, ResearchSquare). Certainty rating should be reconsidered upon peer-reviewed publication.

Outcome 5: Prognostic Value

Question: Does eye-tracking performance predict functional recovery or command-following at follow-up in DOC patients?

Key Evidence: Johansson et al. (2025): 62.5% (5/8) of patients with overt tracking at baseline demonstrated command-following at 1-year follow-up.

GRADE Domain	Rating	Justification

X1. Risk of Bias	Serious (−1)	Single study with prognostic follow-up; small subsample (n = 8 with overt tracking); no blinding of outcome assessors; high risk of attrition bias (follow-up rate not reported for full cohort).
X2. Inconsistency	Not assessable (0)	Single study; cannot assess consistency.
X3. Indirectness	Not serious (0)	Direct evidence in target population with relevant prognostic outcome (command-following at 1 year).
X4. Imprecision	Very serious (−2)	Extremely small sample (n = 8 with overt tracking); wide confidence intervals (95% CI: 24.5–91.5% for 62.5% proportion); single-center study; no replication.
X5. Publication Bias	Suspected (−1)	**Rationale: Publication bias is suspected based on: (1) near-absence of null results in the included set — all included studies report positive ET performance metrics; (2) selective outcome reporting risk in small DOC studies where negative findings are less likely to be submitted or accepted; (3) the grey literature search did not identify unpublished studies with null results; (4) no contact with authors of registered but unpublished trials was feasible. Fewer than 10 studies contribute to this outcome; funnel plot analysis is therefore**

		**statistically inappropriate (Sterne et al., 2011). This rating is based on narrative assessment per GRADE guidance.**

Starting Certainty: Observational studies = LOW (⨁⨁○○)

Downgrades: Risk of bias (−1), Very serious imprecision (−2), Publication bias (−1)

Upgrades: None

Final Certainty: ⨁○○○ VERY LOW

Part 3: GRADE Domain Definitions and Application

X1. Risk of Bias

Definition: Limitations in study design or execution that may bias the results.

Assessment Criteria:

- Blinding of assessors (ET and CRS-R conducted independently?)
- Randomization of stimulus presentation order
- Standardization of assessment protocols
- Completeness of outcome data
- Selective outcome reporting

Application in This Review:

• Serious risk of bias (−1) applied to all outcomes due to universal lack of blinding

• Additional concerns: non-randomized stimulus order, variable CRS-R protocols, small single-center studies

---

X2. Inconsistency

Definition: Unexplained heterogeneity or variability in results across studies.

Assessment Criteria:

• Consistency in direction of effect

• Overlap of confidence intervals

• Statistical heterogeneity (I^2 statistic, if meta-analysis conducted)

• Explainability of heterogeneity by subgroup or sensitivity analyses

Application in This Review:

• Not serious (0) for most outcomes: direction of effect consistent despite variability in magnitude

• Heterogeneity in absolute detection rates explained by stimulus type, population severity, and technology differences

• No formal meta-analysis conducted due to heterogeneity in methods and outcomes

---

X3. Indirectness

Definition: Differences between the evidence and the review question in terms of population, intervention, comparator, or outcome (PICO).

Assessment Criteria:

• Population: Are study participants representative of the target population (prolonged DOC)?

• Intervention: Is eye-tracking technology as implemented in studies representative of clinical use?

• Comparator: Is CRS-R the appropriate reference standard?

• Outcome: Are measured outcomes directly relevant to clinical decision-making?

Application in This Review:

• Not serious (0) for all outcomes: studies directly address the review question in the target population with relevant interventions and comparators

---

X4. Imprecision

Definition: Uncertainty in effect estimates due to small sample sizes or wide confidence intervals.

Assessment Criteria:

• Sample size (total number of patients and events)

• Width of confidence intervals

• Whether confidence intervals include both clinically important benefit and harm

• Optimal information size (OIS) considerations

Application in This Review:

• Serious imprecision (−1) applied to most outcomes due to small sample sizes (n = 6–88 per study) and lack of reported confidence intervals

• Very serious imprecision (−2) applied to prognostic outcome due to extremely small sample (n = 8 with overt tracking)

X5. Publication Bias

Definition: Systematic underestimation or overestimation of effects due to selective publication of studies with positive or statistically significant results.

Assessment Criteria:

• Funnel plot asymmetry (if ≥10 studies available)

• Grey literature search results

• Trial registry searches for unpublished studies

• Narrative assessment of likelihood of publication bias

Application in This Review:

• Suspected (−1) for four outcomes (Detection Rate, Diagnostic Discrimination, Affective Salience, Prognostic Value): **near-absence of null results; selective outcome reporting**

risk in small DOC studies; grey literature search did not identify unpublished null-result studies; fewer than 10 studies per outcome preclude funnel plot analysis

• Unlikely serious (0) for Sensitivity/Specificity outcome: **single prospective diagnostic accuracy study with pre-specified outcomes reporting both sensitivity and specificity; however, preprint status introduces uncertainty**

Part 4: Rationale for Overall Certainty Ratings

Outcome 1: Detection Rate — LOW Certainty (⨁⨁○○)

Starting point: Observational studies = LOW (⨁⨁○○)

Downgrades:

1. Risk of bias (−1): Lack of blinding in all studies; CRS-R and ET assessments not conducted independently

2. Imprecision (−1): Small sample sizes; wide variability in detection rates (18–97%); confidence intervals not reported

3. Publication bias (−1): **Near-absence of null results; selective outcome reporting risk; grey literature search yielded no unpublished null-result studies; funnel plot analysis not feasible (fewer than 10 studies)**

Upgrades: None

Final certainty: LOW (⨁⨁○○) — Limited confidence in the effect estimate; true effect may be substantially different

Interpretation: Eye-tracking consistently detects more visual responses than CRS-R alone, but the magnitude of this advantage is uncertain due to methodological limitations and small sample sizes.

Outcome 2: Diagnostic Discrimination — LOW Certainty (⨁⨁○○)

Starting point: Observational studies = LOW (⨁⨁○○)

Downgrades:

1. Risk of bias (−1): Lack of blinding; non-randomized stimulus presentation order; risk of carryover effects

2. Imprecision (−1): Small sample sizes; limited replication of stimulus comparisons; confidence intervals not reported

3. Publication bias (−1): **Near-absence of null results; selective outcome reporting risk; grey literature search yielded no unpublished null-result studies; funnel plot analysis not feasible (fewer than 10 studies)**

Upgrades: None

Final certainty: LOW (⨁⨁○○) — Limited confidence in the stimulus hierarchy; true effect may be substantially different

Interpretation: Mirror stimuli consistently achieve highest detection rates, but the precise magnitude of differences between stimulus types is uncertain.

Outcome 3: Affective Salience Response — LOW Certainty (⊕⊕○○)

Starting point: Observational studies = LOW (⊕⊕○○)

Downgrades:

1. Risk of bias (−1): Lack of blinding; subjective classification of "affective" vs. "neutral" stimuli; inconsistent operationalization

2. Imprecision (−1): Small sample sizes; wide variability in effect magnitude; confidence intervals not consistently reported

3. Publication bias (−1): **Near-absence of null results; selective outcome reporting risk; grey literature search yielded no unpublished null-result studies; funnel plot analysis not feasible (fewer than 10 studies)**

Upgrades: None

Final certainty: LOW (⊕⊕○○) — Limited confidence in the affective salience effect; true effect may be substantially different

Interpretation: Affectively salient stimuli elicit stronger tracking responses in MCS patients, but the magnitude and clinical significance of this effect are uncertain.

Outcome 4: Sensitivity/Specificity — VERY LOW Certainty (⨁○○○)

Starting point: Observational studies = LOW (⨁⨁○○)

Downgrades:

1. Risk of bias (−1): Single preprint study; blinding status unclear; reference standard applied by same team; risk of incorporation bias

2. Imprecision (−1): Single study with n = 88; wide confidence intervals for specificity (70.8–97.6%); no replication

No downgrade for publication bias: **Single prospective diagnostic accuracy study with pre-specified outcomes reporting both sensitivity and specificity; however, preprint status introduces uncertainty about reporting completeness**

Upgrades: None

Final certainty: VERY LOW (⨁○○○) — Very little confidence in the effect estimate; true effect likely substantially different

Interpretation: High diagnostic accuracy estimates are promising but based entirely on a single non-peer-reviewed preprint. Certainty rating should be reconsidered upon peer-reviewed publication and replication.

Outcome 5: Prognostic Value — VERY LOW Certainty (⨁○○○)

Starting point: Observational studies = LOW (⨁⨁○○)

Downgrades:

1. Risk of bias (−1): Single study; small subsample (n = 8 with overt tracking); no blinding of outcome assessors; high risk of attrition bias

2. Very serious imprecision (−2): Extremely small sample (n = 8); wide confidence intervals (95% CI: 24.5–91.5%); single-center study; no replication

3. Publication bias (−1): **Near-absence of null results; selective outcome reporting risk; grey literature search yielded no unpublished null-result studies; funnel plot analysis not feasible (fewer than 10 studies)**

Upgrades: None

Final certainty: VERY LOW (⨁○○○) — Very little confidence in the effect estimate; true effect likely substantially different

Interpretation: Preliminary evidence suggests prognostic value, but the extremely small sample size and lack of replication severely limit confidence. Adequately powered prospective cohort studies with systematic follow-up are urgently needed.

Part 5: Clinical Implications of GRADE Certainty

Clinical Practice

Eye-tracking technology shows consistent and potentially important advantages over CRS-R alone for detecting visual responses in DOC patients. The 2.5-fold increase in detection rate and high sensitivity estimates (88.9%–100%) represent clinically meaningful effects that justify use as a supplementary assessment tool. However, the LOW to VERY LOW certainty means that clinicians should interpret individual patient results cautiously and in the context of comprehensive multimodal assessment.

Recommendations:

- Use eye-tracking as a supplementary tool alongside CRS-R, not as a replacement
- Prioritize mirror stimuli for maximum detection sensitivity
- Consider affectively salient stimuli (familiar faces) for MCS patients
- Interpret results cautiously given methodological limitations and small evidence base

---

Clinical Guidelines

Given the LOW certainty for most outcomes, current evidence supports a conditional (weak) recommendation for eye-tracking as a supplementary tool in DOC assessment, rather than a strong recommendation for routine replacement of CRS-R. This aligns with existing clinical guidance (e.g., European Academy of Neurology guidelines) that recommends multimodal assessment.

Conditional recommendation rationale:

- Consistent direction of benefit across studies
- Clinically meaningful effect sizes (2.5-fold detection increase)
- Low risk of harm from supplementary assessment
- BUT: Low certainty due to methodological limitations and small sample sizes

Research Priorities

The VERY LOW certainty for sensitivity, specificity, and prognostic value highlights critical research gaps. The following studies are urgently needed to increase certainty and support stronger clinical recommendations:

Research Gap	Current Limitation	Recommended Study Design
1. Diagnostic accuracy study	Single preprint (Lee et al., 2024); no replication; blinding unclear	Prospective diagnostic accuracy study, n > 100, double-blind design, independent reference standard (CRS-R by blinded assessor), pre-specified diagnostic thresholds
2. Blinded assessment protocol	Blinding absent in all current studies; introduces performance and detection bias	Double-blind design: ET assessor blinded to clinical diagnosis; CRS-R assessor blinded to ET results
3. Standardized reference standard	CRS-R variability across raters and settings; multiple CRS-R assessments needed	Three independent CRS-R assessments per patient; inter-rater reliability ≥0.80
4. Longitudinal prognostic study	Only 1 study with systematic follow-up (n = 8 with overt tracking)	Prospective cohort, n > 100, 6–12 months follow-up, pre-specified outcomes (command-following, functional independence, quality of life)
5. Technology standardization	Heterogeneity of devices prevents pooling; no head-to-head comparisons	Comparative study of 2–3 ET systems in same patients using standardized protocol
6. Systematic confounder control	Arousal, medication, visual impairment inconsistently controlled	Structured assessment of confounders; time-of-day

		standardization; ophthalmological screening

Publication Bias Assessment Methodology

Following GRADE guidance (Guyatt et al., 2008), publication bias was assessed narratively for all outcomes, as the number of studies contributing to each outcome was fewer than ten, rendering funnel plot asymmetry tests statistically unreliable (Sterne et al., 2011). Assessment considered: (a) the proportion of included studies reporting null or negative findings (very low across all outcomes); (b) known reporting biases in small-sample diagnostic accuracy studies in the DOC field; (c) the grey literature search results; and (d) the absence of registered but unpublished studies identified through trial registry searches. These factors collectively support the 'Suspected' publication bias rating for four of five outcomes.

References

1. Guyatt, G.H.; Oxman, A.D.; Vist, G.E.; Kunz, R.; Falck-Ytter, Y.; Alonso-Coello, P.; Schünemann, H.J. (2008). GRADE: An emerging consensus on rating quality of evidence and strength of recommendations. BMJ, 336(7650), 924–926. https://doi.org/10.1136/bmj.39489.470347.AD

2. Sterne, J.A.C.; Sutton, A.J.; Ioannidis, J.P.A.; Terrin, N.; Jones, D.R.; Lau, J.; Carpenter, J.; Rücker, G.; Harbord, R.M.; Schmid, C.H.; et al. (2011). Recommendations for examining and interpreting funnel plot asymmetry in meta-analyses of randomised controlled trials. BMJ, 343, d4002. https://doi.org/10.1136/bmj.d4002
