# Supplementary material for: Eye-Tracking Assessment in Patients with Disorders of Consciousness: A Systematic Review
Source: Brain Sci. 2026 May 30;16(6):590. doi: 10.3390/brainsci16060590 (PMC13297577; doi:10.3390/brainsci16060590)
Supplement: Supplementary file 1 [file brainsci-16-00590-s001.zip › Supplementary File S1.pdf]

Supplementary File 2

Eye-Tracking Assessment in Patients with Disorders of Consciousness: A Systematic Review

Estraneo A., Marcello L., Mancino F., De Feo A., Soricelli A., Franzese M., Cavaliere C.

Brain Sciences (MDPI), 2026

Complete Boolean Search Strings

PubMed Search String

Database: PubMed (National Library of Medicine)

Date of search: [INSERT DATE]

Records retrieved: [INSERT N]

("eye tracking"[MeSH Terms] OR "eye tracking"[tiab] OR "eye movement"[tiab] OR
"gaze tracking"[tiab] OR "visual tracking"[tiab] OR "oculomotor"[tiab] OR
"eye-tracking"[tiab] OR "gaze-tracking"[tiab] OR "visual pursuit"[tiab])
AND
("disorder of consciousness"[tiab] OR "disorders of consciousness"[tiab] OR
"vegetative state"[tiab] OR "unresponsive wakefulness syndrome"[tiab] OR
"minimally conscious state"[tiab] OR "VS/UWS"[tiab] OR "MCS"[tiab] OR
"prolonged disorder of consciousness"[tiab] OR "chronic disorder of consciousness"[tiab] OR
"coma"[MeSH Terms] OR "persistent vegetative state"[MeSH Terms] OR
"consciousness disorders"[MeSH Terms])
AND
("diagnosis"[tiab] OR "diagnostic accuracy"[tiab] OR "detection"[tiab] OR
"assessment"[tiab] OR "evaluation"[tiab] OR "classification"[tiab] OR
"sensitivity"[tiab] OR "specificity"[tiab] OR "prognosis"[tiab] OR
"prognostic"[tiab])

Filters applied: English language; publication date 2000–2025; humans

---

Google Scholar Search String

Database: Google Scholar

Date of search: [INSERT DATE]

Records retrieved: [INSERT N]

"eye tracking" OR "eye movement" OR "gaze tracking" OR "visual tracking"

AND

"disorder of consciousness" OR "vegetative state" OR "unresponsive wakefulness syndrome"

OR "minimally conscious state" OR "MCS" OR "VS/UWS"

AND

"diagnostic accuracy" OR "detection" OR "assessment" OR "CRS-R" OR "Coma Recovery Scale"

Filters applied: Publication date 2000–2025; exclude citations and patents

---

SciSpace Search String

Database: SciSpace (formerly Typeset)

Date of search: [INSERT DATE]

Records retrieved: [INSERT N]

("eye tracking" OR "eye-tracking" OR "gaze tracking" OR "visual pursuit" OR "oculomotor assessment") AND ("disorders of consciousness" OR "vegetative state" OR "minimally conscious state" OR "unresponsive wakefulness syndrome" OR "prolonged DOC") AND ("diagnosis" OR "detection" OR "CRS-R" OR "Coma Recovery Scale" OR "diagnostic accuracy")

Filters applied: Peer-reviewed journals; 2000–2025

Institutional Library / Grey Literature Search String

Database: Institutional library databases (EBSCO, Scopus subset, OpenDOAR repositories)

Date of search: [INSERT DATE]

Records retrieved: [INSERT N]

TI: ("eye tracking" OR "gaze tracking" OR "visual tracking") AND

AB: ("disorder of consciousness" OR "vegetative state" OR "minimally conscious state") AND

AB: ("diagnosis" OR "assessment" OR "detection" OR "CRS-R")

Filters applied: English language; 2000–2025; full text available

---

MeSH Headings Used (PubMed)

**MeSH Term**	**Scope Note**
Eye Tracking	Recording of eye movements for research/clinical purposes
Consciousness Disorders	Includes VS/UWS and MCS
Persistent Vegetative State	Prolonged absence of awareness
Coma	Unarousable unresponsiveness
Diagnostic Techniques, Neurological	Clinical neurological assessment tools
Coma Recovery Scale	Behavioural assessment scale for DOC
