# Supplementary material for: Eye-Tracking Assessment in Patients with Disorders of Consciousness: A Systematic Review
Source: Brain Sci. 2026 May 30;16(6):590. doi: 10.3390/brainsci16060590 (PMC13297577; doi:10.3390/brainsci16060590)
Supplement: Supplementary file 1 [file brainsci-16-00590-s001.zip › Supplementary Table S1.pdf]

Appendix A — PRISMA 2020 Checklist for Systematic Review

Manuscript Title: Eye-Tracking Assessment in Patients with Disorders of Consciousness: A Systematic Review

Authors: Estraneo A., Marcello L., Mancino F., De Feo A., Soricelli A., Franzese M., and Cavaliere C.

PRISMA 2020 Checklist

**Section/Topic**	**Item #**	**Checklist Item**	**Location in Manuscript**	**Reported**
TITLE				
Title	1	Identify the report as a systematic review.	Title page	Yes
ABSTRACT				
Abstract	2	See the PRISMA 2020 for Abstracts checklist.	Abstract (lines 32-55)	Yes
INTRODUCTION				
Rationale	3	Describe the rationale for the review in the context of existing knowledge.	Introduction (lines 56-139)	Yes
Objectives	4	Provide an explicit statement of the objective(s) or question(s) the review addresses.	Introduction (lines 130-139)	Yes
METHODS				
Eligibility criteria	5	Specify the	Methods,	Yes

		inclusion and exclusion criteria for the review and how studies were grouped for the syntheses.	Section 2.2 (lines 164-180)	
Information sources	6	Specify all databases, registers, websites, organisations, reference lists and other sources searched or consulted to identify studies. Specify the date when each source was last searched or consulted.	Methods, Section 2.1 (lines 140-163)	Yes
Search strategy	7	Present the full search strategies for all databases, registers and websites, including any filters and limits used.	**Complete Boolean search strings with MeSH headings and field tags for all four databases (SciSpace, Google Scholar, PubMed, institutional libraries) are provided in Supplementary File 2.**	Yes
Selection process	8	State the process for selecting studies (e.g., screening, eligibility).	Methods, Section 2.3 (lines 181-195)	Yes
Data collection process	9	State the process for collecting data from reports (e.g., piloted forms, independently, in	Methods, Section 2.4 (lines 196-210)	Yes

		duplicate).		
Data items	10a	List and define all outcomes for which data were sought. Specify whether all results that were compatible with each outcome domain in each study were sought (e.g., for all measures, time points, analyses), and if not, the methods used to decide which results to collect.	Methods, Section 2.4 (lines 196-210); Table 1	Yes
	10b	List and define all other variables for which data were sought (e.g., participant and intervention characteristics, funding sources). Describe any assumptions made about any missing or unclear information.	Methods, Section 2.4 (lines 196-210); Table 1	Yes
Study risk of bias assessment	11	Specify the methods used to assess risk of bias in the included studies, including details of the tool(s) used, how many reviewers assessed each study and whether they worked independently, and if applicable, details of automation tools	Methods, Section 2.5 (lines 211-248); Supplementary Appendix D	Yes

		used in the process.		
Effect measures	12	Specify for each outcome the effect measure(s) (e.g., risk ratio, mean difference) used in the synthesis or presentation of results.	Methods, Section 2.6 (lines 249-270)	Yes
Synthesis methods	13a	Describe the processes used to decide which studies were eligible for each synthesis (e.g., tabulating the study intervention characteristics and comparing against the planned groups for each synthesis (item 5)).	Methods, Section 2.6 (lines 249-270)	Yes
	13b	Describe any methods required to prepare the data for presentation or synthesis, such as handling of missing summary statistics, or data conversions.	Methods, Section 2.6 (lines 249-270)	Yes
	13c	Describe any methods used to tabulate or visually display results of individual studies and syntheses.	Methods, Section 2.6 (lines 249-270); Figures 1-5	Yes
	13d	Describe any methods used to synthesize results and provide a rationale for the choice(s). If meta-analysis was performed, describe the	Methods, Section 2.6 (lines 249-270); narrative synthesis approach described	Yes

		model(s), method(s) to identify the presence and extent of statistical heterogeneity, and software package(s) used.		
	13e	Describe any methods used to explore possible causes of heterogeneity among study results (e.g., subgroup analysis, meta-regression).	Methods, Section 2.6 (lines 249-270); heterogeneity discussed narratively	Yes
	13f	Describe any sensitivity analyses conducted to assess robustness of the synthesized results.	Not applicable; insufficient studies for formal sensitivity analysis	N/A
Reporting bias assessment	14	Describe any methods used to assess risk of bias due to missing results in a synthesis (arising from reporting biases).	Methods, Section 2.5 (lines 211-248); Appendix B (GRADE assessment includes publication bias domain)	Yes
Certainty assessment	15	Describe any methods used to assess certainty (or confidence) in the body of evidence for an outcome.	Methods, Section 2.7 (lines 271-301); Appendix B (GRADE Evidence Profile)	Yes
RESULTS				
Study selection	16a	Describe the results of the search and selection process, from the number of records identified in the search to the	Results, Section 3.1 (lines 302-318); Figure 1 (PRISMA flow diagram)	Yes

		number of studies included in the review, ideally using a flow diagram.		
	16b	Cite studies that might appear to meet the inclusion criteria, but which were excluded, and explain why they were excluded.	**Two reviewers (A.E. and C.C.) independently screened all records. Inter-rater agreement: κ = 0.89 for full-text inclusion stage. Disagreements resolved by consensus.** Results, Section 3.1 (lines 302-318); Supplementary Appendix E (List of Excluded Studies with Reasons)	Yes
Study characteristics	17	Cite each included study and present its characteristics.	**Full-text PDFs unavailable for two studies (Johansson et al., 2021; Aklepi et al., 2024); data extracted from abstracts and DOI metadata. This limitation is acknowledged in Results and Discussion.** Results, Section 3.2 (lines 319-331); Table 1	Yes

Risk of bias in studies	18	Present assessments of risk of bias for each included study.	Results, Section 3.3 (lines 332-350); Figure 2	Yes
Results of individual studies	19	For all outcomes, present, for each study: (a) summary statistics for each group (where appropriate) and (b) an effect estimate and its precision (e.g., confidence/credible interval), ideally using structured tables or plots.	Results, Sections 3.4-3.7 (lines 351-450); Table 2, Table 3, Figures 3-5	Yes
Results of syntheses	20a	For each synthesis, briefly summarise the characteristics and risk of bias among contributing studies.	Results, Sections 3.4-3.7 (lines 351-450)	Yes
	20b	Present results of all statistical syntheses conducted. If meta-analysis was done, present for each the summary estimate and its precision (e.g., confidence/credible interval) and measures of statistical heterogeneity. If comparing groups, describe the direction of the effect.	Results, Sections 3.4-3.7 (lines 351-450); narrative synthesis presented due to heterogeneity	Yes
	20c	Present results of all investigations of possible causes of heterogeneity among study	Results, Section 3.4 (lines 351-380); heterogeneity discussed	Yes

		results.	narratively	
	20d	Present results of all sensitivity analyses conducted to assess the robustness of the synthesized results.	Not applicable; insufficient studies for formal sensitivity analysis	N/A
Reporting biases	21	Present assessments of risk of bias due to missing results (arising from reporting biases) for each synthesis assessed.	Results, Section 3.8 (lines 451-470); Appendix B (GRADE Evidence Profile)	Yes
Certainty of evidence	22	Present assessments of certainty (or confidence) in the body of evidence for each outcome assessed.	**Publication bias ratings are based on narrative assessment of selective outcome reporting risk and near-absence of null results in included studies. Fewer than 10 studies contribute to any single outcome; funnel plot analysis is therefore not appropriate. Narrative justification provided in Results and Supplementary File 3.** Results, Section 3.8 (lines 451-470); Appendix B (GRADE	Yes

			Evidence Profile)	
DISCUSSION				
Discussion	23a	Provide a general interpretation of the results in the context of other evidence.	Discussion, Section 4.1 (lines 471-550)	Yes
	23b	Discuss any limitations of the evidence included in the review.	Discussion, Section 4.1 (Limitations subsection, lines 520-550)	Yes
	23c	Discuss any limitations of the review processes used.	Discussion, Section 4.1 (Limitations subsection, lines 520-550)	Yes
	23d	Discuss implications of the results for practice, policy, and future research.	Discussion, Section 4.2 (lines 551-580); Conclusions (lines 581-600)	Yes
OTHER INFORMATION				
Registration and protocol	24a	Provide registration information for the review, including register name and registration number, or state that the review was not registered.	**This review was not prospectively registered in PROSPERO. Eligibility criteria and primary outcomes were pre-specified prior to database searching. The absence of registration is acknowledged as a limitation in Methods and Discussion.**	Yes
	24b	Indicate where the	No formal	Yes

		review protocol can be accessed, or state that a protocol was not prepared.	protocol was prepared beyond internal planning documents.	
	24c	Describe and explain any amendments to information provided at registration or in the protocol.	Not applicable (no registration or formal protocol).	N/A
Support	25	Describe sources of financial or non-financial support for the review, and the role of the funders or sponsors in the review.	Funding statement (lines 601-605)	Yes
Competing interests	26	Declare any competing interests of review authors.	Conflicts of Interest statement (lines 606-610)	Yes
Availability of data, code, and other materials	27	Report which of the following are publicly available and where they can be found: template data collection forms; data extracted from included studies; data used for all analyses; analytic code; any other materials used in the review.	Data Availability Statement (lines 611-620); Supplementary Appendices A-F	Yes
